# Definitive Radiation Therapy for Merkel Cell Carcinoma Misdiagnosed as a Metastatic Tumor: A Case Report

**DOI:** 10.7759/cureus.5483

**Published:** 2019-08-25

**Authors:** Toshiaki Matsui, Takahiro Oike, Katsuyuki Shirai, Tatsuya Ohno

**Affiliations:** 1 Department of Radiation Oncology, Gunma University Graduate School of Medicine, Maebashi, JPN; 2 Department of Radiology, Saitama Medical Center, Jichi Medical University, Omiya, JPN

**Keywords:** merkel cell carcinoma, radiation therapy, diagnosis, pathology, case report

## Abstract

Merkel cell carcinoma (MCC) is a rare and aggressive cutaneous neuroendocrine neoplasia. Surgical resection is the first-line therapeutic option, and radiation therapy is an alternative treatment for inoperable cases. Herein, we report a case of primary MCC (cT2N0M0, stage IIA) of the head and neck region. This case was misdiagnosed as a metastatic tumor and referred to the department of radiation oncology for palliative irradiation. Additional immunohistochemical analysis confirmed the diagnosis of MCC, and the tumor was treated with definitive radiation therapy (66 Gy in 33 fractions), leading to complete in-field control. This case indicates that even in patients with suspected metastatic tumors referred for palliative treatment, patient characteristics and pathology should be carefully examined to avoid missing potentially controllable primary tumors. In such cases, MCC, although rare, should be included in the differential diagnosis of head and neck lesions.

## Introduction

Merkel cell carcinoma (MCC) is cutaneous neuroendocrine neoplasia characterized by a highly aggressive behavior [1-2]. MCC is an extremely rare type of cancer, with an incidence of 0.6 cases per 100,000 person-years in the United States [3]. However, the incidence of MCC is increasing rapidly, highlighting the importance of the appropriate clinical management of this disease [1]. Surgery is the first-line therapeutic option in the treatment of primary MCCs [1]. Radiation therapy is a potentially definitive treatment alternative to surgery because MCCs show high radiosensitivity [4]. Herein, we report a case of primary MCC of the head and neck region. This case was misdiagnosed as a metastatic tumor by four clinical departments and referred to our department of radiation oncology for palliative irradiation. Additional pathological analyses performed at our institution led to the diagnosis of MCC. The patient was treated with definitive radiation therapy, which resulted in complete in-field control.

## Case presentation

A 90-year-old Japanese woman diagnosed with a metastatic tumor in the left cheek was referred to our department of radiation oncology from the department of urology for the purpose of palliative irradiation on March 1 of 20XX. She was followed up by the department of urology for nine years for a tumor in the right kidney (1.7 cm in size). The follow-up consisted of computed tomography (CT) without biopsy because of the stable nature of the tumor. She also received hemodialysis for six years because of chronic renal dysfunction.

In the summer of 20XX-1, the patient noticed a tiny tumor in her left cheek. After January of 20XX, the tumor showed rapid growth. On February 21 of 20XX, the tumor was 2 cm in diameter with a violaceous appearance, and a biopsy of the tumor was performed. The immunohistochemical (IHC) analysis showed that the biopsy specimen was positive for chromogranin, synaptophysin, and CD56 and negative for thyroid transcription factor-1 (TTF-1), S-100, and human melanin black-45 (HMB-45). The pathologist reported it as a metastatic neuroendocrine tumor of unknown origin. Based on the pathology report, the urologist diagnosed the tumor as a metastatic tumor derived from the right kidney. The patient was referred to the departments of dermatology and plastic surgery. The dermatologist and the plastic surgeon agreed with the diagnosis made by the pathologist and the urologist and considered the tumor inoperable because of its extremely rapid-growing nature. The patient was referred for palliative radiation therapy.

**Figure 1:**
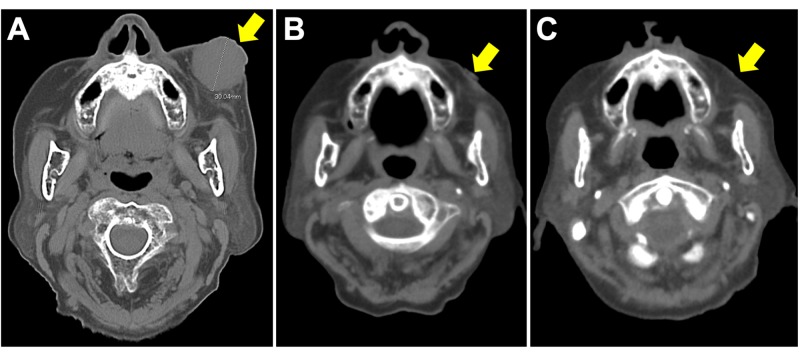
Computed tomography images of a Merkel cell carcinoma of the left cheek (A) Before initiation of radiation therapy; (B) at the completion of radiation therapy; (C) three months after initiation of radiation therapy; arrows indicate the tumor site.

At the time of presentation to our department of radiation oncology, the tumor was 3 cm in diameter. CT images showed that the tumor was almost completely spherical (Figure 1). The CT examination showed no lesions indicative of lymph node- or distant-metastases in the regions from the head through the pelvis; the patient’s tumor in the right kidney had remained stable for nine years. The characteristics of the patient and the tumor were consistent with MCC, although the IHC analysis was insufficient for the differential diagnosis of MCC (see Discussion for details). We, therefore, consulted with a second pathologist with experience in dermatology, who performed additional IHC staining against cytokeratin-20 (CK-20). The results showed that the biopsy specimen was positive for CK-20 with a paranuclear dot-like pattern typical of MCC [1]. Taken together, these findings led us to diagnose the patient as primary MCC of the left cheek, cT2N0M0, stage IIA [5].

**Figure 2:**
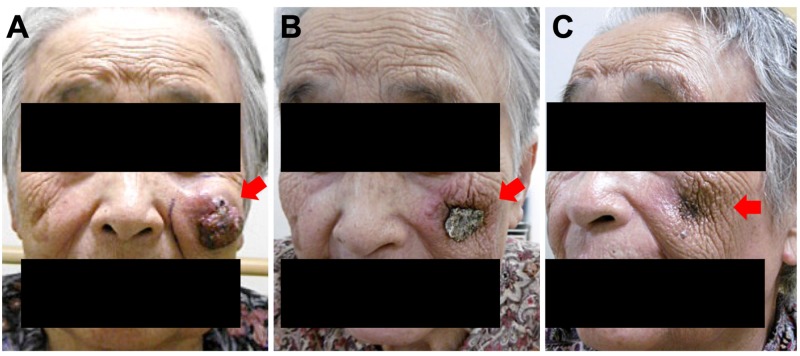
Appearance of the Merkel cell carcinoma of the left cheek (A) Before initiation of radiation therapy; (B) at the completion of radiation therapy; (C) three months after initiation of radiation therapy; arrows indicate the tumor.

Based on the diagnosis of primary MCC, the tumor was treated with definitive (but not palliative) radiation therapy at a total dose of 66 Gy in 33 fractions. Radiation (9 MeV electrons) was delivered to the gross tumor with a margin of 2-3 cm using a 5-mm-thick tissue-equivalent material (commonly referred to as bolus). The radiation field was modified to avoid the left eye. The tumor showed rapid remission during radiation therapy; the energy was kept the same during the course with an intention to cover tumor bed as the tumor shrinks. On the day of completion of radiation therapy, the tumor achieved almost complete remission (Figure 2). The patient experienced Grade 1 dermatitis (as assessed by the Common Terminology Criteria for Adverse Effects, version 4.0). At three months after initiation of radiation therapy, in-field tumor control was achieved with no evidence of lymph node- or distant-metastases in the regions from the head through the pelvis. The skin surface remained without defects. The patient is being followed up by CT every three months up to three years, and every six months thereafter to carefully monitor for regional and distant metastases [1].

## Discussion

Radiation oncologists receive referrals for palliative radiation of metastatic tumors on a daily basis. The doses utilized for palliative radiation are insufficient for tumor control (e.g., 30 Gy in 10 fractions or 8 Gy in 1 fraction). This case of MCC was misdiagnosed as a metastatic tumor by four clinical departments and then referred to our department for palliative treatment. However, after the differential diagnosis of MCC, the patient was treated with definitive radiation therapy that led to complete tumor remission without severe toxicities at least for three months after initiation of radiation therapy. The lesson learned from this case may be that the characteristics of a tumor, in terms of presentation, epidemiology, and pathology, should be carefully considered even in cases referred for palliative treatment of metastatic tumors to avoid missing potentially controllable primary tumors. MCC, although rare, should be included in the differential diagnosis of metastatic tumors in the head and neck regions.

MCC presents as a rapidly growing, firm, red to violaceous nodule localized on the skin exposed to ultraviolet (UV) radiation [2]. It is common in patients with suppressed immune status including those undergoing chronic hemodialysis [6-9]. The incidence of MCC increases markedly in patients aged >70 years, which is attributed to the burden of chronic mutagenic UV exposure and age-associated diminished immuno-surveillance [6]. The present case was consistent with the characteristics of MCC described above, which led us to perform additional IHC analysis for the differential diagnosis of MCC.

Pathologically, MCC belongs to the family of small round cell tumors. The diagnosis of MCC is made on the basis of detailed IHC analysis of biopsy samples [6]. Positive CK-20 staining is observed in 75-100% of MCC cases, and a paranuclear dot-like staining pattern is typical, whereas CK-20 positivity is rarely observed in small cell carcinoma of the lung and of other organs [1]. By contrast, TTF-1 is positive in >80% of small cell carcinomas and never positive in MCC [1]. Therefore, analysis of both CK-20 and TTF-1 is important to differentiate MCC from other small cell carcinomas. Neuroendocrine markers (e.g., chromogranin, synaptophysin, and CD56), melanoma markers (e.g., S-100), and lymphoma markers, e.g., leukocyte common antigen (LCA), support the differential diagnosis in equivocal cases [1,3]. In the present case, IHC staining against CK-20 had not been performed at the time of referral to our department; therefore, the possibility of MCC needed to be considered.

Surgical resection is the first-line therapeutic option in the treatment of primary MCCs [1]. However, in many patients, surgery is not the optimal approach because of the following reasons: (i) MCC occurs frequently in the head and neck regions, in which resection of the tumor with adequate surgical margins and without functional and cosmetic sequelae is difficult; and (ii) MCC is prevalent in the elderly, in which general anesthesia, surgery, and hospitalization represent a high risk. When surgical resection is not feasible, definitive radiation therapy is considered as an alternative first-line treatment for MCC [3,10]. In such situations, 60-66 Gy administered at the conventional fractionation of 1.8 to 2 Gy per treatment is recommended [1]. The clinical target volume typically encompasses the gross tumor with a 3-5 cm margin, and bolus is used to assure sufficient radiation delivery to the full thickness of the skin and underlying fascia [6]. These treatment strategies were applied in the present case. The treatment outcomes of patients receiving definitive radiation therapy for MCC have not been reported in detail because of the limited number of relevant studies and the small sample sizes of existing studies. A meta-analysis of locoregional MCCs treated with definitive radiation therapy reported an in-field control rate of 75% to 85% during a follow-up period of 23.7 ± 21.9 months [10]. These data taken together with the present case indicate the efficacy of definitive radiation therapy for the treatment of inoperable MCC. Sentinel lymph node biopsy (SLNB) is recommended based on data showing that 30% to 38% of patients with MCC are positive for the test [1]. In MCC patients who do not undergo SLNB, prophylactic irradiation with 46-50 Gy to the nodal basin is recommended [1]. However, the survival benefit of SLNB and that of prophylactic irradiation for clinically node-negative cases are unclear [1,11]. In addition, nodal dissection or prophylactic irradiation may increase the risk for lymphedema [12]. Furthermore, radiation therapy can be used at a later time to salvage regional failures [12]. In the present case, prophylactic irradiation to the nodal basin was not performed upon informed consent by the patient and the family member.

## Conclusions

We presented a case of MCC misdiagnosed by four clinical departments as a metastatic tumor and referred for palliative radiation therapy. Careful consideration of the case presentation and additional IHC analysis led to the differential diagnosis of MCC, and in-field control of the bulky tumor was achieved through definitive radiation therapy. Even in cases diagnosed as metastatic tumors and referred for palliative radiation, the patient characteristics and pathology should be carefully considered to avoid missing potentially controllable primary tumors. MCC, although rare, should be included in the differential diagnosis of metastatic tumors in the head and neck regions.
